# Ultrahigh Responsivity and Detectivity Graphene–Perovskite Hybrid Phototransistors by Sequential Vapor Deposition

**DOI:** 10.1038/srep46281

**Published:** 2017-04-19

**Authors:** Po-Han Chang, Shang-Yi Liu, Yu-Bing Lan, Yi-Chen Tsai, Xue-Qian You, Chia-Shuo Li, Kuo-You Huang, Ang-Sheng Chou, Tsung-Chin Cheng, Juen-Kai Wang, Chih-I Wu

**Affiliations:** 1 Graduate Institute of Photonics and Optoelectronics, National Taiwan University, Taipei, 106, Taiwan (R.O.C.); 2 Center for Condensed Matter Sciences, National Taiwan University, Taipei, 10617, Taiwan (R.O.C.); 3 Institute of Atomic and Molecular Sciences, Academia Sinica, Taipei, 10617, Taiwan (R.O.C.); 4 Department of Electrical Engineering, National Taiwan University, Taipei, 106, Taiwan (R.O.C.)

## Abstract

In this work, graphene-methylammonium lead iodide (MAPbI_3_) perovskite hybrid phototransistors fabricated by sequential vapor deposition are demonstrated. Ultrahigh responsivity of 1.73 × 10^7^ A W^−1^ and detectivity of 2 × 10^15^ Jones are achieved, with extremely high effective quantum efficiencies of about 10^8^% in the visible range (450–700 nm). This excellent performance is attributed to the ultra-flat perovskite films grown by vapor deposition on the graphene sheets. The hybrid structure of graphene covered with uniform perovskite has high exciton separation ability under light exposure, and thus efficiently generates photocurrents. This paper presents photoluminescence (PL) images along with statistical analysis used to study the photo-induced exciton behavior. Both uniform and dramatic PL intensity quenching has been observed over entire measured regions, consistently demonstrating excellent exciton separation in the devices.

Graphene, a promising material with excellent electronic and optoelectronic properties, is regarded as a future potential candidate in many technology fields^1^,^2^,^3^,^4^. The first single-layer lattice plane of graphite, graphene, was discovered in 2004^5^. In particular, its extremely high carrier mobility of ∼200,000 cm^2^ V^−1^ s^−1^ and ballistic transport characteristics provide the potential of achieving high-speed field effect transistors (FETs)^6^,^7^, which could replace conventional silicon-based FETs in the future^1^,^8^. For applications in light detection, the first ultrafast graphene photodetector with extremely high operational bandwidth has been demonstrated, enabled by graphene’s outstanding electrical properties and gapless nature^9^,^10^. However, the light absorption of monolayer graphene is only 2.3%^11^. The responsivity of pristine graphene photodetectors is limited to ∼10^−3^ A W^−1^ due to the poor light absorption cross-section in active regions, short photon-generated carrier lifetime with the scale of tens of picoseconds, and the absence of a gain mechanism^9^,^10^,^12^. To overcome these problems, introducing semiconducting light absorbers to enhance optical absorption and to prevent fast exciton recombination in graphene is regarded as a practicable approach for fabricating high-performance graphene-based photodetectors. For instance, utilizing graphene as highly transparent electrodes on semiconductors^13^,^14^,^15^ or metal oxide nanostructures^16^,^17^ has been proposed for metal-free Schottky junction photodiodes. Relatively high responsivity of up to 435 mA W^−1^ has been achieved by exploiting graphene-silicon heterojunctions^15^. Although the improvement in responsivity is substantial, the performance is still not sufficient for light detection at low-intensity illumination. In 2012, novel graphene-based phototransistors with graphene/colloidal quantum dots (QDs) hybrid channels reached sufficiently high performance levels for low light detection^18^. This device prototype with ultrahigh gain (∼10^8^) and responsivity (∼10^7^ A W^−1^) has been considered the promising candidate for extremely low-intensity imaging sensors^18^. After that, reports were published in rapid succession on highly sensitive photodetectors or photo-memory devices produced by hybridizing graphene with other light absorbers, like QDs^19^,^20^, two-dimensional materials^21^, or organic semiconductors^22^,^23^.

Recently, mixed organic-inorganic halide perovskite has received great attention for its exceptional optoelectronic properties, such as excellent optical absorption characteristics, long exciton diffusion length (∼100 nm for methylammonium lead iodide (MAPbI_3_) perovskite) and high carrier mobility^24^. This material has been widely applied in photovoltaics^24^,^25^,^26^,^27^, light-emitting devices^27^,^28^,^29^, and photodetectors^30^,^31^,^32^,^33^. For photodetection, Lee *et al*. first proposed graphene-MAPbI_3_ perovskite hybrid photodetectors with high responsivity of 180 A W^−1^ and effective quantum efficiency of 5 × 10^4^%^32^. Later, Wang *et al*. also demonstrated graphene–CH_3_NH_3_PbBr_2_I hybrid photodetectors^33^, and reported an ultrahigh responsivity of ∼6 × 10^5^ A W^−1^ and gain of ∼10^9^. The gain mechanism of these ultrasensitive photodetectors is based on the photogating effect^18^,^32^,^33^. That is, the specific type of carriers, separated from the photo-excited excitons at the graphene/perovskite interface, will transfer to the graphene channel to change its conductivity and thus produce photocurrents by applying a drain voltage, while the opposite type of carriers will be trapped in the perovskite films with a relatively long lifetime. However, Lee’s group revealed that the perovskite films on graphene formed via the simple spin-coating process showed a non-uniform film thickness profile with peak regions up to 480 nm and valley regions of about 100 nm^32^. They also demonstrated the integrated photoluminescence (PL) intensity mapping images of MAPbI_3_ perovskite films spin-coated onto graphene (see the supporting information of ref. 32). According to the absorption length at the wavelength of 550 nm and the carrier diffusion length of MAPbI_3_ perovskite, which are both approximately 100 nm^24^,^25^,^32^,^34^, only a few excitons originating from the 480-nm-thick perovskite peak region can reach the graphene/perovskite interface and be separated to generate photo-carriers in graphene (schematically illustrated in Supplementary Fig. S1), causing the high exciton radiative recombination rate (i.e., high local PL intensity)^32^. In contrast, most of the excitons originating from the 100-nm-thick perovskite valley region will effectively result in being separated (schematically illustrated in Supplementary Fig. S2), leading to high quenching efficiency and low local PL intensity^32^. The integrated PL images of MAPbI_3_ perovskite films spin-coated on graphene exhibited a non-uniform distribution of PL intensities^32^, and the regions with high local PL intensities inefficiently contributed to the photocurrents of the devices, indicating that the heterostructure of graphene hybridized with bumpy and non-flat perovskite reduces the quenching and photocurrent generation efficiencies of hybrid phototransistors. Furthermore, Wang’s group combined CH_3_NH_3_PbBr_2_I nanoparticles with graphene to produce active regions of hybrid phototransistors by the spin-coating process^33^. However, numerous local apertures were found in the perovskite, where the short exciton lifetime and low optical absorption will lead to weak photocurrents in the phototransistors. It was pointed out that it seems technically difficult to fabricate uniform perovskite films on graphene using the traditional spin-coating process^32^,^33^, and the non-uniform film thickness and incomplete coverage of perovskite will be the main drawbacks that lower the responsivity of the devices. Spina *et al*. reported high-performance phototransistors by hybridizing MAPbI_3_ nanowires (NWs) and graphene by slit coating, and a responsivity of 2.6 × 10^6^ A W^−1^ was achieved^35^. Nevertheless, the relatively long response time of ∼5 s greatly restricted the operational speed of the devices. The lag was ascribed to numerous and long-lived traps in the nanowire films^35^,^36^.

Dual-source vapor deposition is regarded as an appropriate process to fabricate high-quality perovskite films with superior uniformity, and is able to realize efficient planar photovoltaic devices with high power conversion efficiency (PCE) (over 15%) by co-evaporating two precursors of lead halides and methylammonium iodides (MAI)^37^,^38^,^39^. However, the small molecular weight of MAI makes it difficult to accurately control the deposition rate through the co-evaporation process in a vacuum chamber^40^. The alternative approach is sequential deposition consisting of a layer-by-layer deposition process with one precursor at a time, with a deposition rate that is much easier to control and monitor^40^,^41^. In this work, we demonstrated high-performance graphene–MAPbI_3_ perovskite hybrid phototransistors produced by sequential vapor deposition with an ultrahigh responsivity of 1.73 × 10^7^ A W^−1^ and detectivity of 2 × 10^15^ Jones under low-intensity white light illumination. The photodetector performance is better than other similar graphene–MAPbI_3_ perovskite hybrid devices reported to date. This work realized extremely high effective quantum efficiencies of up to 10^8^% in the visible range (450–700 nm). This excellent device performance is attributed to the ultra-flat perovskite films grown on the graphene, capable of efficient light harvesting and exciton separation. Under light exposure, the PL images along with statistical analysis imply the efficient generation of photocurrents in the active regions. Moreover, the device exhibits a faster response time of ∼879 ms (corresponding to ∼70% drop in photocurrent), as compared to previously reported graphene/MAPbI_3_ NWs phototransistors^35^. The ultrasensitive graphene–perovskite hybrid phototransistors are capable of very low-intensity light detection.

## Results

**Figure 1:**
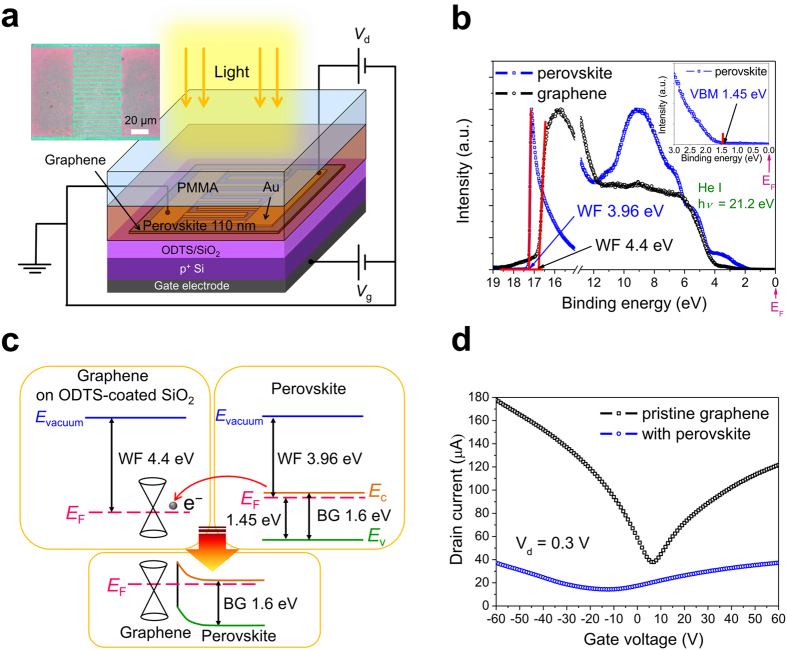
(**a**) Schematic illustration of the graphene–perovskite hybrid phototransistor. Inset shows the top-view optical microscopy image of the device. (**b**) He I UPS spectra of graphene on ODTS-coated SiO_2_ substrates and MAPbI_3_ perovskite on ITO glass. Inset shows the valance band edge along with VBM of perovskite. (**c**) Band diagrams of graphene and perovskite obtained from UPS spectra. The schematic diagram of the band bending at graphene/perovskite interfaces is illustrated below. (**d**) Transfer curves of the GFET before and after perovskite deposition (*V*_d_ = 0.3 V). The channel length and width were set to be identical (both 3 μm).

The schematic illustration of the graphene–MAPbI_3_ perovskite hybrid phototransistor fabricated in this work is shown in Fig. 1a. The underlying graphene field effect transistor (GFET) was established on self-assembled monolayer (SAM)-modified SiO_2_ substrates, which consists of 300 nm thick SiO_2_ grown via the thermal oxidation process on heavily doped p-type silicon substrates and Octadecyltrichlorosilane (ODTS) on the as-grown SiO_2_. The SAM forming process produces depressed electric doping effects^42^ and significant improvement in electrical properties of transferred graphene^43^, provided by the hydrophobic surface formed by SAMs^44^. The channel length and width of this GFET with interdigital electrodes were 3 and 1200 μm, respectively. A MAPbI_3_ perovskite film was deposited as the active layer of the graphene–perovskite hybrid phototransistor with a thickness of 110 nm. To prevent contamination from ambient moisture and oxygen, poly(methyl methacrylate) (PMMA) was spin-coated on the entire surface of the device for passivation^45^. To study the crystalline structures of MAPbI_3_ perovskite, X-ray diffraction (XRD) analysis was adopted (Supplementary Fig. S3). The peaks located at 2*θ* = 14.29°, 28.55°, 32°, 40.73°, and 43.27° correspond to the (110), (220), (310), (224), and (314) planes of MAPbI_3_ perovskite. The (110) and (220) preferred orientations were observed, which is consistent with the reported results for MAPbI_3_ perovskite films^46^ and confirms that the compact crystalline MAPbI_3_ perovskite films were achieved by sequential vapor deposition. The stoichiometry of MAPbI_3_ perovskite was obtained by X-ray photoemission spectroscopy (XPS) using Mg K*α* radiation (*hν* = 1253.6 eV). The I/Pb atomic ratio was measured to be 2.9, which is close to the desired value (3.0).

The valence-band electronic structures of graphene and MAPbI_3_ perovskite were first studied via ultraviolet photoelectron spectroscopy (UPS) to investigate the electronic characteristics of graphene/perovskite heterostructure. The UPS spectra of the graphene on ODTS-coated SiO_2_ substrates and MAPbI_3_ perovskite (~200 nm) deposited on indium tin oxide-coated glass (ITO glass) substrates using He I radiation are shown in Fig. 1b. The secondary electron onset, which is evaluated by the linear extrapolation of the high binding-energy cutoff region, represents the vacuum level of the measured sample^47^. The Fermi level in the UPS spectrum is set to be zero as the reference energy point. The presented values of binding energies are all relative to the Fermi level. Thus, the work function (WF) of a sample is determined from the secondary-electron onset as WF = *hν* − *E*_onset_, where *hν* and *E*_onset_ are the photon energy of He I radiation (21.2 eV) and the binding energy corresponding to secondary-electron onset, respectively. The onset of the graphene sample was measured to be 16.8 eV, corresponding to a WF of 4.4 eV. The onset, WF, and valence band maximum (VBM) (refer to the inset in Fig. 1b) of the MAPbI_3_ perovskite sample were found to be 17.24 eV, 3.96 eV, and 1.45 eV, respectively, which were consistent with the reported values for MAPbI_3_ perovskite films^48^. Figure 1c shows the individual band structures of the graphene on ODTS-coated SiO_2_ substrates and MAPbI_3_ perovskite according to the UPS spectra. The WF mismatch between graphene and MAPbI_3_ perovskite indicates electron transfer from MAPbI_3_ perovskite to graphene, and thus builds an internal electric field at the interface, aiding the separation of photo-generated excitons. Figure 1d expresses the transfer curves of the GFET without and with MAPbI_3_ perovskite. The channel length and width are both 3 μm. The ambipolar characteristics originating from semi-metallic or gapless nature of graphene yield switchable active carrier types by altering gate voltages in GFETs^5^,^6^. The transfer curves of the pristine GFET and that covered with MAPbI_3_ perovskite both show underlying V shapes consisting of hole conduction branches (gate voltage < charge neutrality points (CNP)) and electron conduction branches (gate voltage > CNP). The pristine GFET exhibits nearly intrinsic characteristics (CNP ∼7 V), while a negative shift of CNP from 7 V to −12 V is observed after MAPbI_3_ perovskite deposition. This negative shift implies a strong n-type doping effect due to electrons transferred from MAPbI_3_ perovskite, consistent with the energetic alignment of the interface band structure obtained by the UPS spectra. Furthermore, the hole and electron mobility of graphene decrease from 1231 cm^2 ^V^−1^ s^−1^ to 212 cm^2^ V^−1^ s^−1^ and from 894 cm^2^ V^−1^ s^−1^ to 141 cm^2^ V^−1^ s^−1^, respectively, after depositing MAPbI_3_ perovskite due to external defects introduced to the surface of graphene during the deposition process.

**Figure 2:**
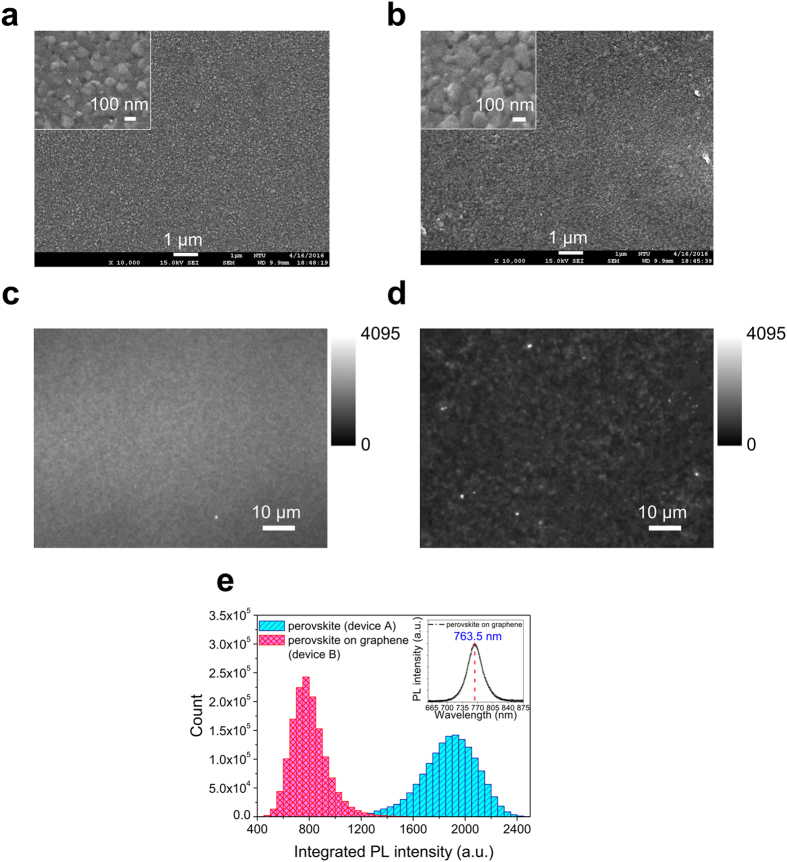
The SEM images of perovskite films deposited on (**a**) ODTS-coated SiO_2_ substrates and (**b**) graphene. Insets show the locally magnified images within the original area. The PL images of perovskite films deposited on (**c**) ODTS-coated SiO_2_ substrates and (**d**) graphene with the grey value scale on the right. (**e**) Histograms of PL intensity images analyzed from (**c**) and (**d**). Inset shows the PL spectrum of perovskite deposited on graphene.

The film morphologies of MAPbI_3_ perovskite were studied on two devices, MAPbI_3_ perovskite/ODTS/SiO_2_ and MAPbI_3_ perovskite/graphene/ODTS/SiO_2_, namely, devices A and B in the following section. The selected film thickness of the perovskite was 110 nm. The scanning electron microscope (SEM) images of device A and B are shown in Fig. 2a,b, respectively. The two SEM images reveal comparable morphology in the MAPbI_3_ perovskite, and both exhibit the superior uniformity and full coverage obtained from sequential vapor deposition. An atomic force microscope (AFM) was used to verify the surface roughness of device A and B (Supplementary Fig. S4). The results express the extremely low root mean square roughness of device A and B, measured as 4.5 nm and 4.9 nm, respectively, which also shows a slight difference in uniformity of the two devices. PL measurements were carried out to investigate the photo-physical behavior in the graphene–perovskite heterostructure. The integrated PL images of devices A and B are shown in Fig. 2c,d, respectively. In device B, both overall and dramatic PL intensity depressions are observed over the measured regions. This phenomenon originates from when the photo-generated excitons are separated near graphene/perovskite interfaces, ending up with non-radiative quenching, which is observed by dark regions in the PL images by integrating the decay of the PL intensity^32^. The statistical distribution of integrated PL intensities from device A (Fig. 2c) and device B (Fig. 2d) are shown in Fig. 2e. The significantly decreased PL mean intensity and standard deviation of PL intensity are observed. Unlike the highly non-uniform PL intensity distribution of the devices formed by the solution process^32^, the heterostructure of graphene covered with ultra-flat MAPbI_3_ perovskite films with a thickness of 110 nm fabricated by sequential vapor deposition clearly demonstrates the uniform and dramatic quenching across the entire graphene/perovskite interface. As a result, a high efficiency of exciton separation in the whole hybrid system was achieved in this study, leading to high photocurrents (i.e., high responsivities) and an extremely low radiative recombination rate in perovskite bulks. In addition, some regions of the integrated PL maps in device B show a few bright spots, indicating radiative recombination with a low quenching efficiency, probably due to incomplete transformation residues of MAPbI_3_ perovskite via sequential vapor deposition. The inset in Fig. 2e shows the PL spectrum of the MAPbI_3_ perovskite film deposited on graphene, showing its peak located at 763.5 nm with a corresponding optical bandgap of ∼1.6 eV, which indicates that the active zone of the perovskite–graphene hybrid phototransistors we demonstrated in this work mostly lie in the MAPbI_3_ perovskite bulk.

**Figure 3:**
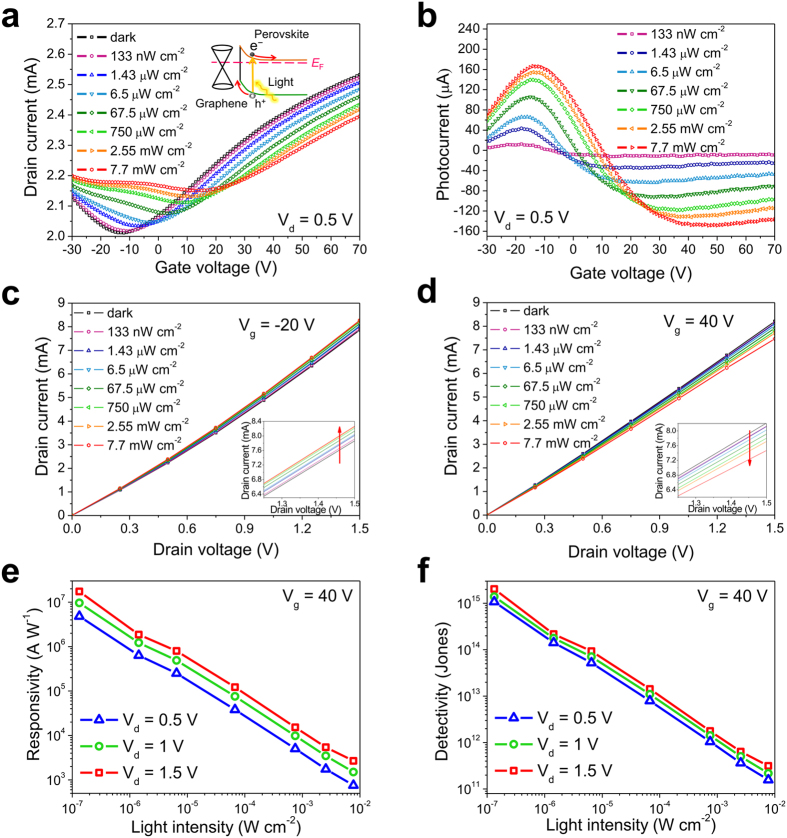
(**a**) The transfer curves of the hybrid phototransistor at a drain voltage of 0.5 V at several selected light intensity levels. A schematic diagram of charge transfer between graphene and perovskite films under light illumination is embedded at the top-right corner. (**b**) The photocurrents of the hybrid phototransistor with respect to gate voltage under several selected light intensity levels. The characteristic curves of the hybrid phototransistor at gate voltages of (**c**) −20 V and (**d**) 40 V under several light intensity levels. Inset shows the expanded region from 1.25 to 1.5 V. (**e**) Responsivity and (**f**) detectivity of the hybrid phototransistor with respect to light intensities at three selected drain voltages.

The device characteristics were first studied through the light-intensity-dependent response under white light irradiation consisting of several selected intensity levels provided by white light-emitting diodes (WLEDs). The light-intensity-dependent transfer curves of the pristine GFET with interdigital electrodes are shown in Supplementary Fig. S5. It shows no photo-response in transfer characteristics of the pristine GFET. Figure 3a expresses the light-intensity-dependent transfer characteristics of the graphene–MAPbI_3_ perovskite hybrid phototransistor at a persistent source-drain voltage bias of 0.5 V, and the corresponding photocurrents (
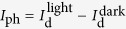
) with respect to gate voltages are shown in Fig. 3b, where 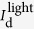 and 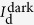 are the drain currents of the phototransistor under light and dark conditions, respectively. The photocurrents of the graphene–perovskite hybrid phototransistor show intensity direction tunable characteristics through applying specific gate voltage under any fixed light intensity. This phenomenon is explained by the schematic illustration of the charge transfer between graphene and perovskite films shown in the inset of Fig. 3a. Charge transfer is the dominant mechanism in the photo-response of the hybrid phototransistor. Since the active region of the phototransistor in this work lies in MAPbI_3_ perovskite, most photo-excited electrons and holes reside in the perovskite layer. These photo-excited holes transferred into graphene produce a photo-induced p-type doping effect as well as a positive CNP shift to graphene. The positive CNP shift varies with light intensity due to the number of photo-excited holes generated, as illustrated in Fig. 3a. When the hybrid phototransistor is operated in the hole conduction region (gate voltage < CNP), the hole concentration in the channel of the phototransistor is raised by transferred photo-induced holes from perovskite with an enhancement in positive photocurrent. In contrast, when the hybrid phototransistor is operated in the electron conduction region (gate voltage > CNP), the transferred photo-induced holes compensate the gate-induced free electrons and the residual electrons generate negative photocurrent in the phototransistor. The tunable zero photo-response is thus reached at a specific gate voltage, which is also related to the number of transferred holes corresponding to a given light intensity. Such gate-controllable photo-response phenomena are important in photodetection^18^. The characteristic curves at a gate voltage of −20 V (in the hole conduction region) and of 40 V (in the electron conduction region) with respect to several selected light intensities are shown in Fig. 3c,d, respectively. The drain current intensity shows a linear correlation with drain voltage regardless of applied gate voltage. Figure 3e shows the responsivities of the graphene–MAPbI_3_ perovskite hybrid phototransistor with respect to light intensities at three selected drain voltages (*V*_d_ = 0.5, 1.0, 1.5 V) when biased with a gate voltage of 40 V. The responsivity (*R*) is defined as |*I*_ph_| × *P*^−1^, where *I*_ph_ and *P* are photocurrent and incident illumination power, respectively. The maximum responsivity of the graphene–MAPbI_3_ perovskite hybrid phototransistor we demonstrated in this work is 1.73 × 10^7^ A W^−1^, achieved at a drain voltage of 1.5 V, gate voltage of 40 V, and low white light intensity of 133 nW cm^−2^. It is approximately ten orders of magnitude higher than that of pristine graphene photodetectors^9^,^10^,^12^.

The ultrahigh responsivity is attributed to the compact and uniform coverage of MAPbI_3_ perovskite formed by sequential vapor deposition. As mentioned previously, the non-uniform film thickness and incomplete coverage of perovskite on graphene channels result in a high bulk recombination rate and low optical absorption cross-section, respectively, both of which decrease the responsivities of the devices. The vapor deposition technique effectively reduces the non-uniformity of the film thickness and the number of tiny apertures^37^,^40^,^41^, and therefore dramatically increases the capability of exciton separation and vertical absorption cross section. With these improvements, more photo-excited excitons are generated and separated near the graphene/perovskite interface, which significantly increases the responsivity in the hybrid phototransistor. Moreover, this hybrid phototransistor exhibits a high carrier mobility (∼200 cm^2^ V^−1^ s^−1^), nearly three orders of magnitude higher than that of MAPbI_3_ perovskite (0.18 cm^2^ V^−1^ s^−1^)^45^. That is, the photo-generated holes transferred into graphene recirculate many times with relatively long lifetime and mean free path, resulting in a high photoconductive gain^18^. The hybrid phototransistor shows excellent ability in exciton separation, and has ultrahigh responsivity, especially at extremely low intensities of incident light. It is worth mentioning that lower responsivity is observed as the incident light power increases. As more excitons are generated, an additional electric field established by photo-induced holes transferred into graphene and the corresponding electrons left in perovskite layers weakens the original internal field near the graphene/perovskite interface built by the Fermi-level alignment in heterostructure. The ability in exciton separation declines with reduced interfacial electric field, leading to decreased photocurrent as the incident light intensity increases.

Another critical parameter for photodetectors is detectivity (D*), which represents the capability of detecting low-level light signals. It is given by D* = (∆*f A*)^1/2^*R/i*_n_, where *R, A*, ∆*f*, and *i*_n_ are the responsivity, active area of a phototransistor, electrical bandwidth, and noise current, respectively^49^,^50^. While the noise current of phototransistors is dominated by the shot noise, the noise current in the shot-noise limit is given by 
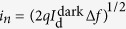
, where *q* and 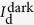 are the value of the elementary charge and the dark drain current of a phototransistor, respectively^50^. Therefore, D* in the shot-noise limit can be calculated by the simple expression: 
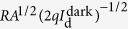
31,49,50, which has been used to estimate the detectivities of phototransistors in previously reported studies^35^,^50^,^51^,^52^,^53^. Figure 3f shows the detectivities of the graphene-MAPbI_3_ perovskite hybrid phototransistor with respect to incident light intensities at the identical drain voltage as in Fig. 3d. The maximum detectivity in the shot-noise limit we demonstrated in this work is 2 × 10^15^ Jones, achieved at a drain voltage of 1.5 V, gate voltage of 40 V, and low white light intensity of 133 nW cm^−2^. This value is several orders of magnitude higher than that of MAPbI_3_ perovskite/graphene devices on polyimide substrates (3 × 10^12^ Jones in the shot-noise limit)^53^ and is comparable to the previously reported value for MAPbI_3_ NWs/graphene devices (~1 × 10^15^ Jones in the shot-noise limit)^35^. The ultrasensitive characteristics we achieved in the hybrid phototransistor enable the detection of exceedingly low-intensity light.

**Figure 4:**
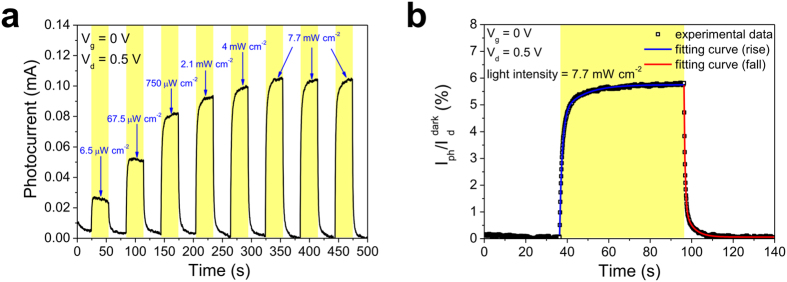
(**a**) The time-dependent photocurrent response with on and off half cycles for 30 s. The illumination intensity of each pulse rises incrementally, and terminates at the ultimate intensity of 7.7 mW cm^−2^. (**b**) The temporal photocurrent response under light intensity of 7.7 mW cm^−2^ and fit with independent exponential functions for on and off half cycles separately.

The time-dependent photocurrent response measurements utilizing WLEDs as the light source were carried out under a drain bias of 0.5 V and zero gate voltage. In Fig. 4a, the input light intensity pulse sequence is composed of on-and-off half cycles for 30 s, and the ultimate light intensity is set to be 7.7 mW cm^−2^. The photocurrents exhibit a drastic rise and fall corresponding to input light pulse switching, and show excellent reproducibility in the last cycles under ultimate light intensity. Figure 4b expresses the temporal photocurrent response fit with independent exponential functions in on and off half cycles separately under an ultimate light intensity of 7.7 mW cm^−2^. The rise in photocurrent is fit with the combination of two exponential functions: 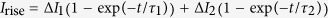
19,22. The short relaxation time constant *τ*_1_ indicating the time duration for photo-induced holes transferring from the perovskite film to graphene is 1.23 s; the long relaxation time constant *τ*_2_ relating to the time duration for charge transfer in the perovskite layer is 10.27 s. Similarly, the fall in photocurrent is fitted with the following function: 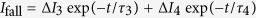
19,22. The short decay time constant *τ*_3_ representing the lifetime of the electrons trapped in the perovskite film is 0.53 s; the long decay time constant *τ*_4_ referring to the time duration for charge transfer and transportation in the perovskite layer is 4.12 s. The fall time (*τ*_fall_) defined by the time taken for a 70% drop in photocurrent is ∼879 ms, which is comparable to the previous reported value for CH_3_NH_3_PbBr_2_I/graphene devices (*τ*_fall_ ∼750 ms)^33^ and much smaller than that of MAPbI_3_ NWs/graphene devices (*τ*_fall_ ∼5 s)^35^. The device performance of our hybrid phototransistors via the sequential vapor deposition technique and other similar graphene–MAPbI_3_ perovskite hybrid devices previously reported are listed by citation in Table 1 for comparison^32^,^33^,^35^,^53^,^54^.

**Figure 5:**
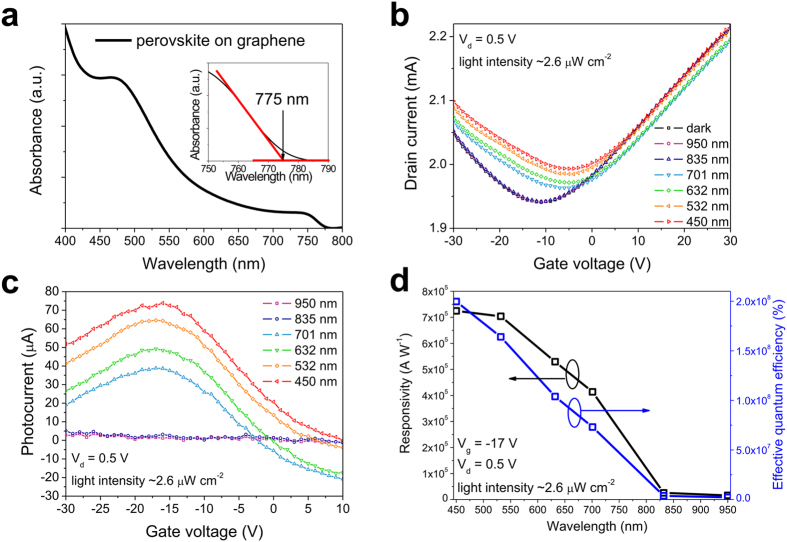
(**a**) The optical absorption spectrum of MAPbI_3_ perovskite deposited on graphene. Inset shows the expanded region from 750 to 790 nm with the absorption edge located at 775 nm. (**b**) The transfer curves of the hybrid phototransistor at several selected illumination wavelengths at a fixed light intensity of ∼2.6 μW cm^−2^ and a drain voltage of 0.5 V. The corresponding photocurrents of the hybrid phototransistor are shown in (**c**). (**d**) Responsivity and effective quantum efficiency of the hybrid phototransistor with respect to illumination wavelength.

The absorption spectrum of the graphene–MAPbI_3_ perovskite hybrid film is shown in Fig. 5a. The absorption edge is at 775 nm (refer to the inset in Fig. 5a) with the associative optical bandgap ∼1.6 eV, which indicates that the MAPbI_3_ perovskite absorbs over the full visible range (400 nm–700 nm). In addition, the absorption characteristic curve shows a larger absorption cross-section on the shorter-wavelength side, which is consistent with the reported absorption features of MAPbI_3_ perovskite^39^. To measure the device characteristics of the hybrid phototransistor operated under illumination at various wavelengths, a high power xenon lamp with a monochromator is utilized as the light source to provide light at several selected wavelengths (950, 835, 701, 632, 532, and 450 nm), and the light intensities are controlled to be ∼2.6 μW cm^−2^ by exploiting neutral density filters (described in the experimental section). Figure 5b shows the transfer curves of the graphene–MAPbI_3_ perovskite hybrid phototransistor at a drain bias of 0.5 V at several selected illumination wavelengths. The transfer characteristics show zero photo-response to incident illumination wavelengths above the absorption edge (775 nm), while a positive shift of CNP is observed at the rest of the selected wavelengths. At incident illumination wavelengths below the absorption edge, the photo-induced holes transferred from perovskite apply a p-type doping effect to graphene, and significantly raise the drain current in the hole conduction region (gate voltage < CNP). The corresponding photocurrents of the hybrid phototransistor transfer curves shown in Fig. 5b are shown in Fig. 5c. The photocurrent of the hybrid phototransistor rises at the blue-shift of illumination, because the stronger light harvesting occurred on the shorter-wavelength side of the visible region correlated to the absorption spectra of perovskite. The responsivities and effective quantum efficiencies of the hybrid phototransistor with respect to illumination wavelength, under gate voltage and drain voltage of −17 V and 0.5 V, respectively, are shown in Fig. 5d. The effective quantum efficiency is defined as *η*_eff_ = *η*_ex_G, where *η*_ex_ and G are the external quantum efficiency and the photoconductive gain of the photodetector, respectively^32^. The effective quantum efficiency is calculated by the expression: *η*_eff_ = *R(hv/q*) × 100, where *R, q*, and *hv* are the responsivity, the value of the elementary charge, and the incident photon energy, respectively. The ultrahigh responsivities (>4 × 10^5^ A W^−1^) and effective quantum efficiencies (∼10^8^%) are achieved under an illumination intensity of ∼2.6 μW cm^−2^ in the visible region (450–700 nm). The effective quantum efficiency shows a negative correlation with the illumination wavelength. The effective quantum efficiency at the wavelength of 532 nm is approximately three orders of magnitude higher than that of hybrid photodetectors reported previously (5 × 10^4^%)^32^.

In conclusion, a high-performance and ultrasensitive graphene–perovskite hybrid phototransistor fabricated by sequential vapor deposition was demonstrated, with its ultrahigh responsivity of 1.73 × 10^7^ A W^−1^ and detectivity of 2 × 10^15^ Jones achieved under low-intensity white light illumination. Extremely high effective quantum efficiencies up to ∼10^8^% in the visible range (450–700 nm) were also realized. This excellent device performance is attributed to the uniform perovskite films grown on the graphene sheets by vapor deposition, which provides the compact heterostructure for efficient light harvesting and exciton separation. The superior sensitivity of the fabricated device is the key to producing novel low-intensity imaging sensors and integrated optoelectronic circuits.

## Methods

### Device Fabrication

The fabrication began with 300 nm thick SiO_2_ grown via a dry oxidation process on heavily doped p-type silicon substrates. Prior to graphene transfer, substrates were first coated with ODTS on SiO_2_^55^. The graphene sheet was grown via a chemical vapor deposition (CVD) process on a copper foil^56^, and was transferred utilizing the PMMA-assisted method to ODTS-modified SiO_2_ substrates as the channels of GFETs. Monolayer graphene was identified by Raman spectroscopy (see Supplementary Fig. S6). The interdigital electrodes (Cr 3 nm/Au 30 nm) with channel length of 3 μm and width of 1200 μm were thermally deposited using standard electron-beam lithography. A PbI_2_ (99.999%, Sigma-Aldrich) film with 65 nm thickness was first thermally deposited onto target substrates in a high vacuum chamber (base pressure ∼10^−6^ Torr), then MAI (>98%, Dyesol) was evaporated in a glove box to form the MAPbI_3_ perovskite (see Supplementary Fig. S7). The transformed perovskite film after cooling was rinsed with anhydrous isopropanol^45^,^57^ (99.5%, Sigma-Aldrich) to remove MAI residues on the top surface, dried and annealed^45^. PMMA (average molecular weight ∼350,000, Sigma-Aldrich) dissolved in anhydrous chlorobenzene (99.8%, Sigma-Aldrich) (10 mg/ml) was spin-coated on the top of the perovskite layer at 3000 r.p.m. for 30 s to improve the device stability^45^.

### Materials Characterizations

The thickness of the MAPbI_3_ perovskite film was assessed utilizing a surface profiler (Alpha-Step IQ, KLA-Tencor). UPS and XPS measurements were carried out with a Physical Electronics Phi5400 system, including an ultrahigh vacuum chamber (base pressure of 10^−10^ Torr). In UPS experiments, the photoelectrons excited by He I radiation (*hν* = 21.2 eV) were collected utilizing a hemispherical analyzer (resolution = 0.05 eV). SEM (JSM-7001F, Joel) and AFM (B1022, NT-MDT) were used to study the surface morphology and roughness of the perovskite films. The absorption spectra and crystalline structures of MAPbI_3_ perovskite were investigated using a UV-Vis-NIR spectrophotometer (V-670, JASCO) and an X-ray diffractometer (X’Pert PRO, PANalytical), respectively. PL experiments were performed to investigate exciton behavior of graphene–perovskite hybrid structures with a home-built microspectroscopic system. For steady-state PL measurements, a CW frequency-doubled diode-pumped Nd:YAG laser emitting at 532 nm served as the excitation source. The laser beam was focused by a 10× objective lens through the fused silica window of the cryostat onto the sample top surface. The emitted PL radiation was collected backward by the same objective lens and sent to a 14-cm spectrometer (MicroHR, Horiba) with a 600 gv mm^−1^ grating plus a thermoelectric-cooled charge-coupled device (Newton 920, Andor) for spectral recording. After spectral calibration, the spectral resolution was 2.63 nm and the spectral error was less than 0.25 nm. The laser irradiation power was set at 12.1 nW, corresponding to an irradiation intensity of 15.4 mW cm^−2^. Typical PL signal acquisition time was 60 seconds. No variation in PL intensity was recognized under extended photoirradiation. For PL imaging measurements, a xenon lamp served as the excitation light source. An optical filter set (U-MWG2, Olympus), including an excitation filter (510–560 nm) and an emission filter (>590 nm), was used to select the excitation and emission wavelength ranges. A 100× objective lens was used, yielding ≦4.3 W cm^−2^ irradiation with a total power of 0.66 mW excitation. A cooled CCD camera (CoolSNAP HQ2, Photometrics) was employed for image capturing. The 10-sec integrated images underwent background substraction to remove dark-count image of the CCD.

### Device Measurements

WLEDs (LSD-1025, Taiwan Fiber Optics) were used as the light sources for photo-response measurements. A high power xenon lamp (ASB-XE-175, Spectral Products) with a monochromator (CM110, Spectral Products) was used to measure the effective quantum efficiency at various wavelengths. The light intensities we selected in this work were 2.57, 2.61, 2.61, 2.58, 2.55, and 2.78 μW cm^−2^ for 950, 835, 701, 632, 532, and 450 nm wavelengths, respectively, controlled by neutral density filters (NDC-100C-4M, Thorlabs). The power meter (Nova II, Ophir) collected the light intensities using a laser measurement sensor (PD300-UV, Ophir) for monochromatic light and a broadband measurement sensor (PD300-BB, Ophir) for white light. The hybrid phototransistors were loaded into a vacuum probe system (base pressure ∼10^−3^ Torr), and their electrical and photo-response characteristics were obtained simultaneously using a Keithley 4200 semiconductor characterization system.

## Additional Information

**How to cite this article:** Chang, P.-H. *et al*. Ultrahigh Responsivity and Detectivity Graphene–Perovskite Hybrid Phototransistors by Sequential Vapor Deposition. *Sci. Rep.* **7**, 46281; doi: 10.1038/srep46281 (2017).

**Publisher's note:** Springer Nature remains neutral with regard to jurisdictional claims in published maps and institutional affiliations.

## Supplementary Material

Supplementary Information

## Figures and Tables

**Table 1:** Comparison of previously reported device performance of the phototransistors based on graphene–perovskite hybrid structures.

Active Materials	Substrate	Maximum responsivity [A W^−1^]	Response time (fall) [ms]	Maximum detectivity [Jones]	Reference
MAPbI__3__ films	ODTS/SiO__2__	180	540 (fitting)	10^9^	32
MAPbBr__2__I island	SiO__2__	6 × 10^^5^^	750 (70% decay)	N/A	33
MAPbI__3__ NWs	SiO__2__	2.6 × 10^^6^^	5000 (70% decay)	~1 × 10^15^ (shot-noise limit)	35
MAPbI__3__ island	SiO__2__	2.1 × 10^^3^^	N/A	N/A	54
MAPbI__3__ films	Polyimide	115	5300 (63% decay)	3 × 10^12^ (shot-noise limit)	53
MAPbI__3__ films	ODTS/SiO__2__	1.73 × 10^^7^^	879 (70% decay)	2 × 10^15^ (shot-noise limit)	This work

(MAPbI_3_: CH_3_NH_3_PbI_3_; MAPbBr_2_I: CH_3_NH_3_PbBr_2_I; NWs: nanowires).
