# Supplementary material for: Ultrahigh Responsivity and Detectivity Graphene–Perovskite Hybrid Phototransistors by Sequential Vapor Deposition
Source: Sci Rep. 2017 Apr 19;7:46281. doi: 10.1038/srep46281 (PMC5395820; doi:10.1038/srep46281)
Supplement: Supplementary Information [file srep46281-s1.pdf]

Supplementary Information

Ultrahigh Responsivity and Detectivity Graphene–Perovskite Hybrid Phototransistors by Sequential Vapor Deposition

Po-Han Chang[1], Shang-Yi Liu[1,+], Yu-Bing Lan[1,2,3], Yi-Chen Tsai[1], Xue-Qian You[1], Chia-Shuo Li[1], Kuo-You Huang[1], Ang-Sheng Chou[1], Tsung-Chin Cheng[1], Juen-Kai Wang[2,3], and Chih-I Wu[1,4,*]

[1]Graduate Institute of Photonics and Optoelectronics, National Taiwan University, Taipei, 106, Taiwan (R.O.C.)

[2]Center for Condensed Matter Sciences, National Taiwan University, Taipei, 10617, Taiwan (R.O.C.)

[3]Institute of Atomic and Molecular Sciences, Academia Sinica, Taipei, 10617, Taiwan (R.O.C.)

[4]Department of Electrical Engineering, National Taiwan University, Taipei, 106, Taiwan (R.O.C.)

[+]these authors contributed equally to this work

[*]Corresponding Author

Email: chihiwu@ntu.edu.tw

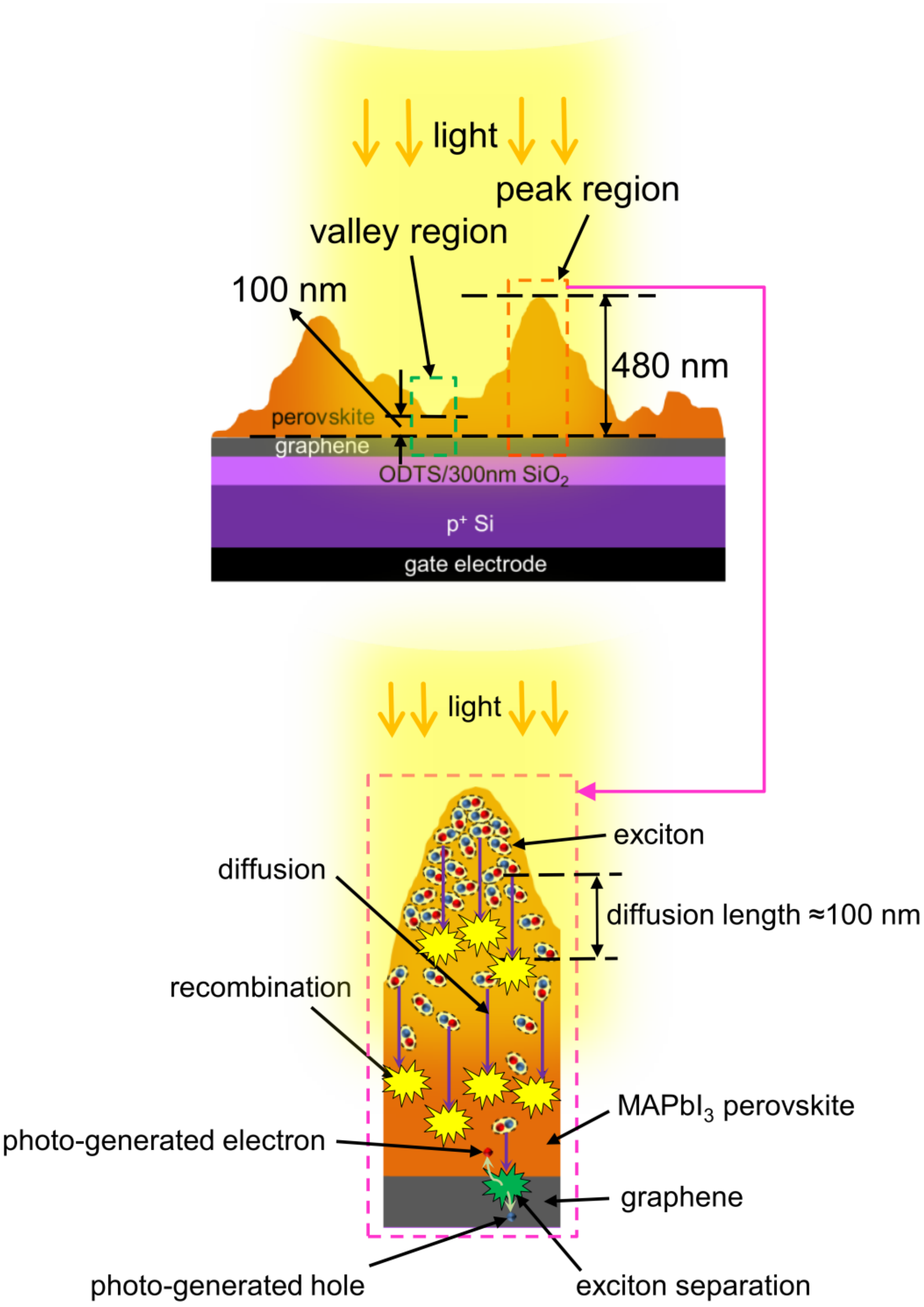

Supplementary Figure S1. The schematic diagram of the photo-excited exciton behavior within the peak regions of graphene–perovskite hybrid films formed by simple spin-coating process.

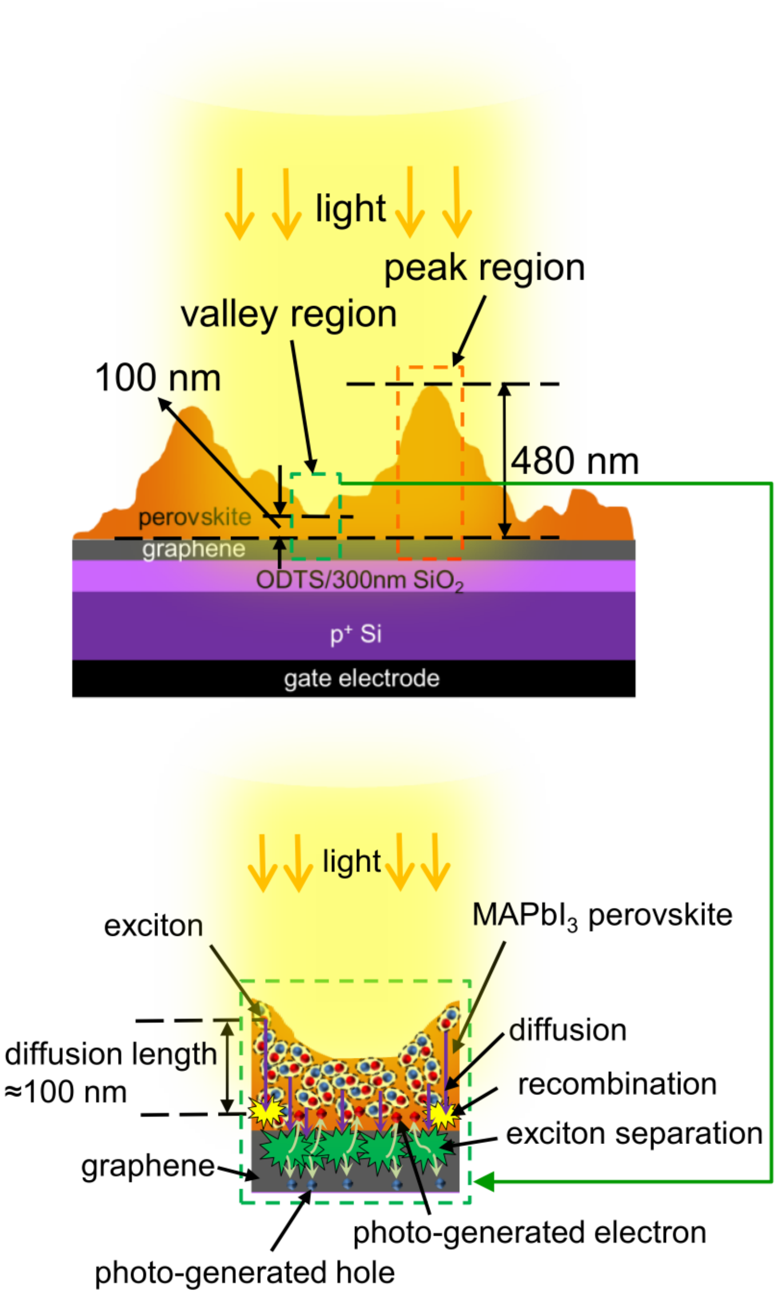

Supplementary Figure S2. The schematic diagram of the photo-excited exciton behavior within the valley regions of graphene–perovskite hybrid films formed by simple spin-coating process.

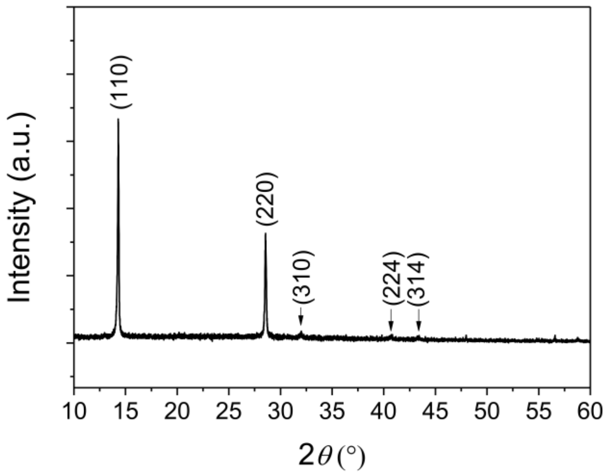

Supplementary Figure S3. X-ray diffraction patterns of $MAPbI_3$ perovskite films formed by sequential vapor deposition.

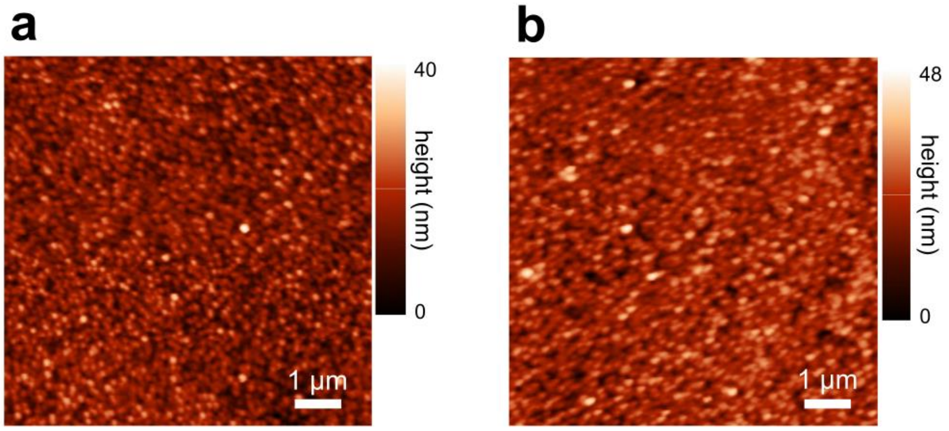

Supplementary Figure S4. The AFM images of perovskite films deposited on (**a**) ODTS-coated SiO_2 substrates and (**b**) graphene.

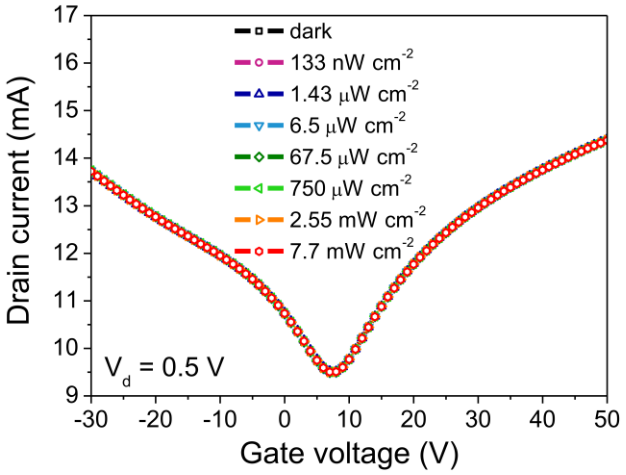

Supplementary Figure S5. The transfer curves of the pristine GFET at a drain voltage of 0.5 V at different light intensities.

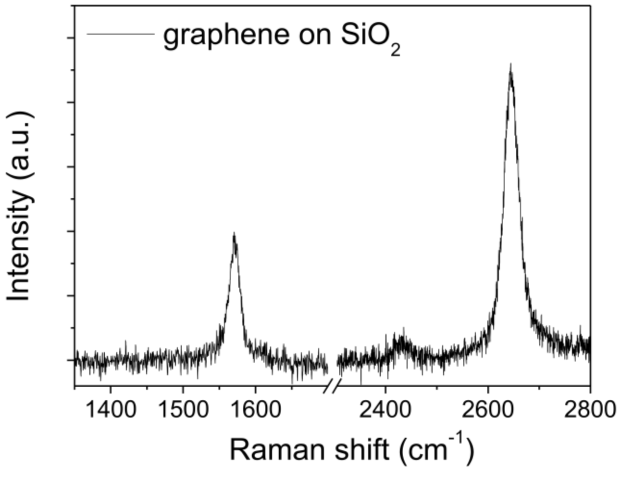

Supplementary Figure S6. Raman spectra of monolayer graphene transferred on SiO_2 substrates.

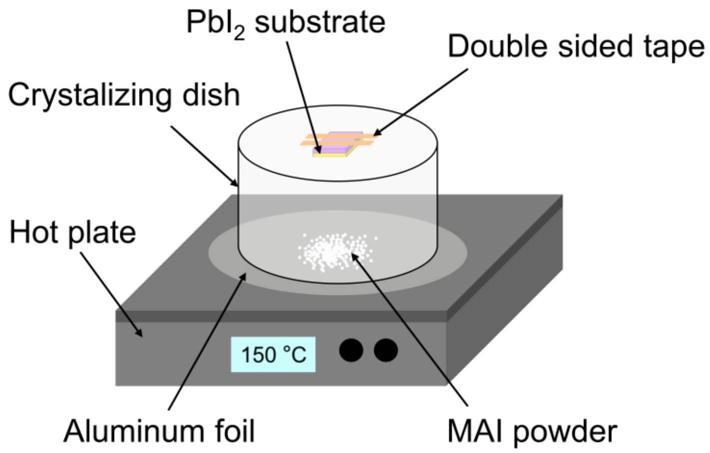

Supplementary Figure S7. The experimental set-up for MAI evaporation. A crystallizing dish was used to fix the samples, which faced the MAI powder spread on an aluminum foil placed on a hot plate of a reaching temperature of 150 ℃.
